# Supplementary material for: Assembling Ultrafine SnO2 Nanoparticles on MIL-101(Cr) Octahedrons for Efficient Fuel Photocatalytic Denitrification
Source: Molecules. 2021 Dec 14;26(24):7566. doi: 10.3390/molecules26247566 (PMC8708904; doi:10.3390/molecules26247566)
Supplement: Supplementary file 1 [file molecules-26-07566-s001.zip › molecules-1493788-supplementary.pdf]

Supporting Information

Assembling ultrafine SnO_2 nanoparticles on MIL-101(Cr) octahedrons for efficient fuel photocatalytic denitrification

Ruowen Liang [a b], Shihui Wang [c], Yi Lu [c], Guiyang Yan [a b d], Zhoujun He [a b],
Yuzhou Xia* [a b], Zhiyu Liang * [a b], Ling Wu * [c],

[a] Department of chemistry, Fujian province university key laboratory of green energy and environment catalysis, Ningde Normal University, Ningde 352100, P. R. China

[b] State key laboratory of photocatalysis on energy and environment, Ningde Normal University, Ningde 352100, P. R. China

[c] State key laboratory of photocatalysis on energy and environment, Fuzhou University, Fuzhou 350002, P. R. China

[d] Xiamen Ocean Vocational College, Xiamen 361000, P. R. China

* Corresponding author: Dr. Yuzhou Xia
Tel: +86-593-2965018; E-mail: yzxia@ndnu.edu.cn

* Corresponding author: Prof. Zhiyu Liang
Tel: +86-593- 0593-2954127; E-mail: lzyfjnu@yeah.net

* Corresponding author: Prof. Ling Wu
Tel: +86-591-83779105; E-mail: wuling@fzu.edu.cn

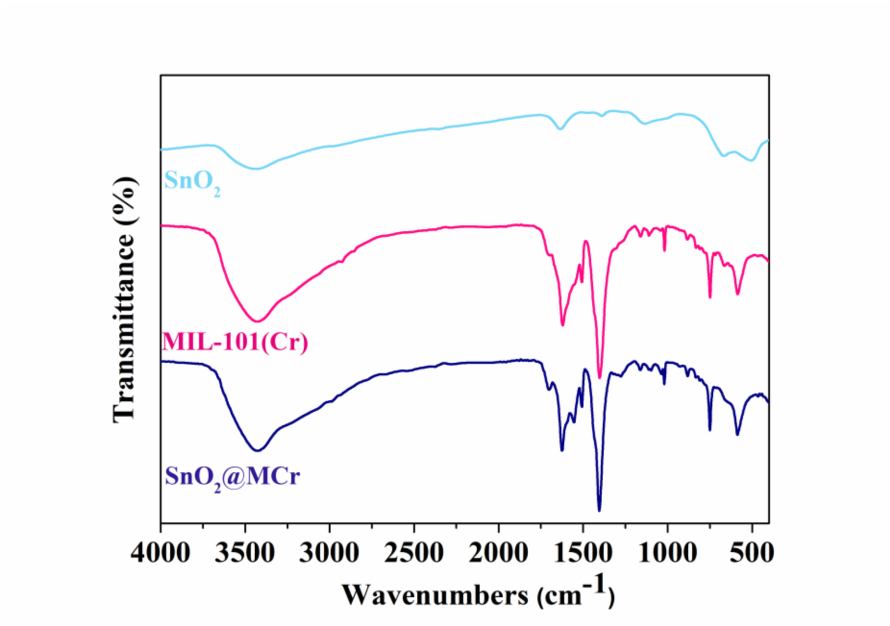

Figure S1. FTIR patterns of MIL-101(Cr), SnO_2 and 20%SnO_2@MCr.

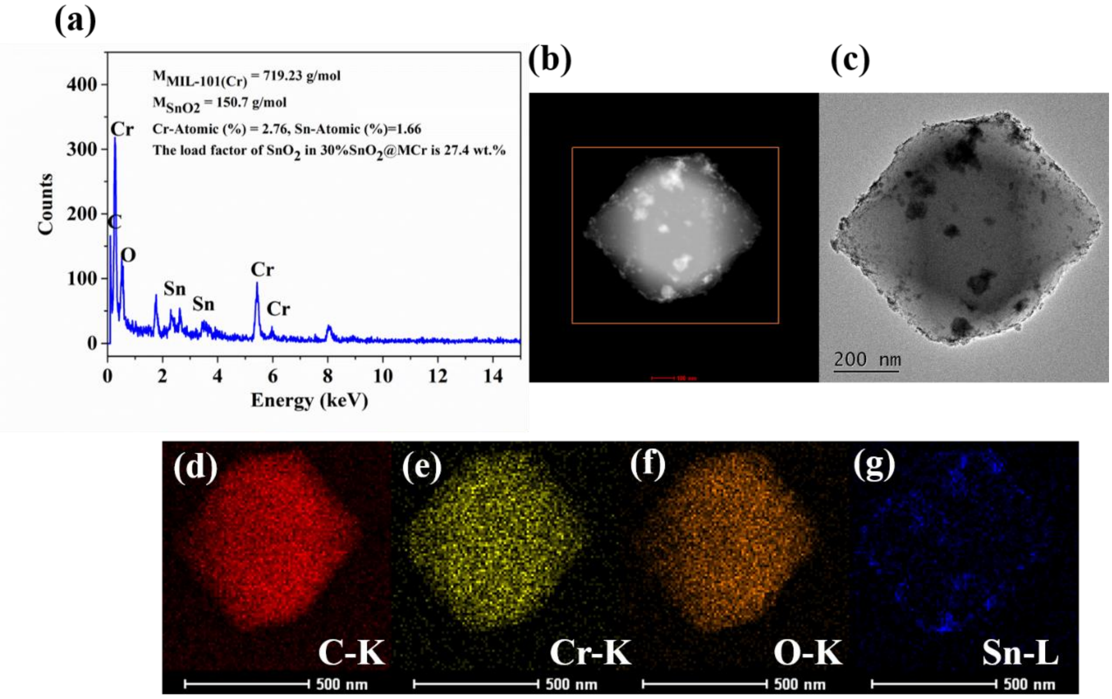

Figure S2. (a) EDS spectrum, (b-g) EDS elemental mappings for the sample of 20%SnO_2@MCr.

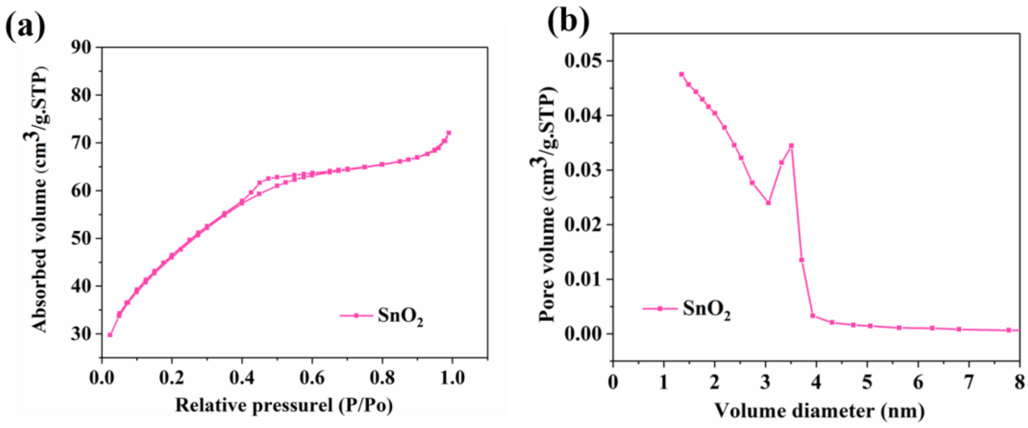

Figure S3. (a) Nitrogen sorption isotherms, (b) pore size distribution of SnO_2.

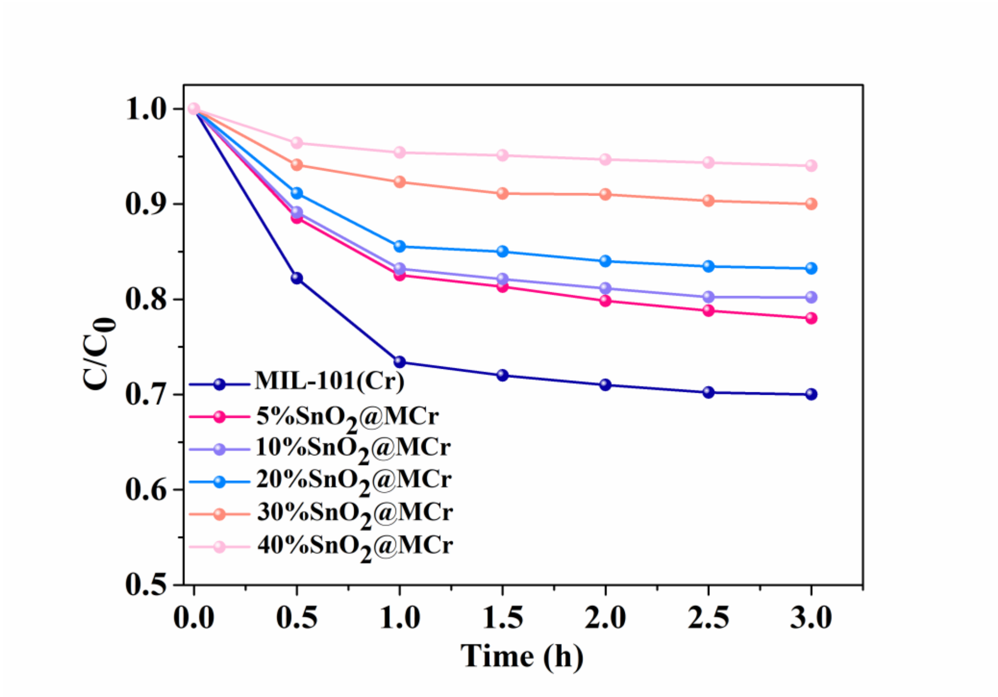

Figure S4. Adsorption capacity of SnO_2@MCr towards pyridine. Reaction conditions: 25 mg of photocatalyst; 50 mL of 100 µg/g pyridine/octane solution.

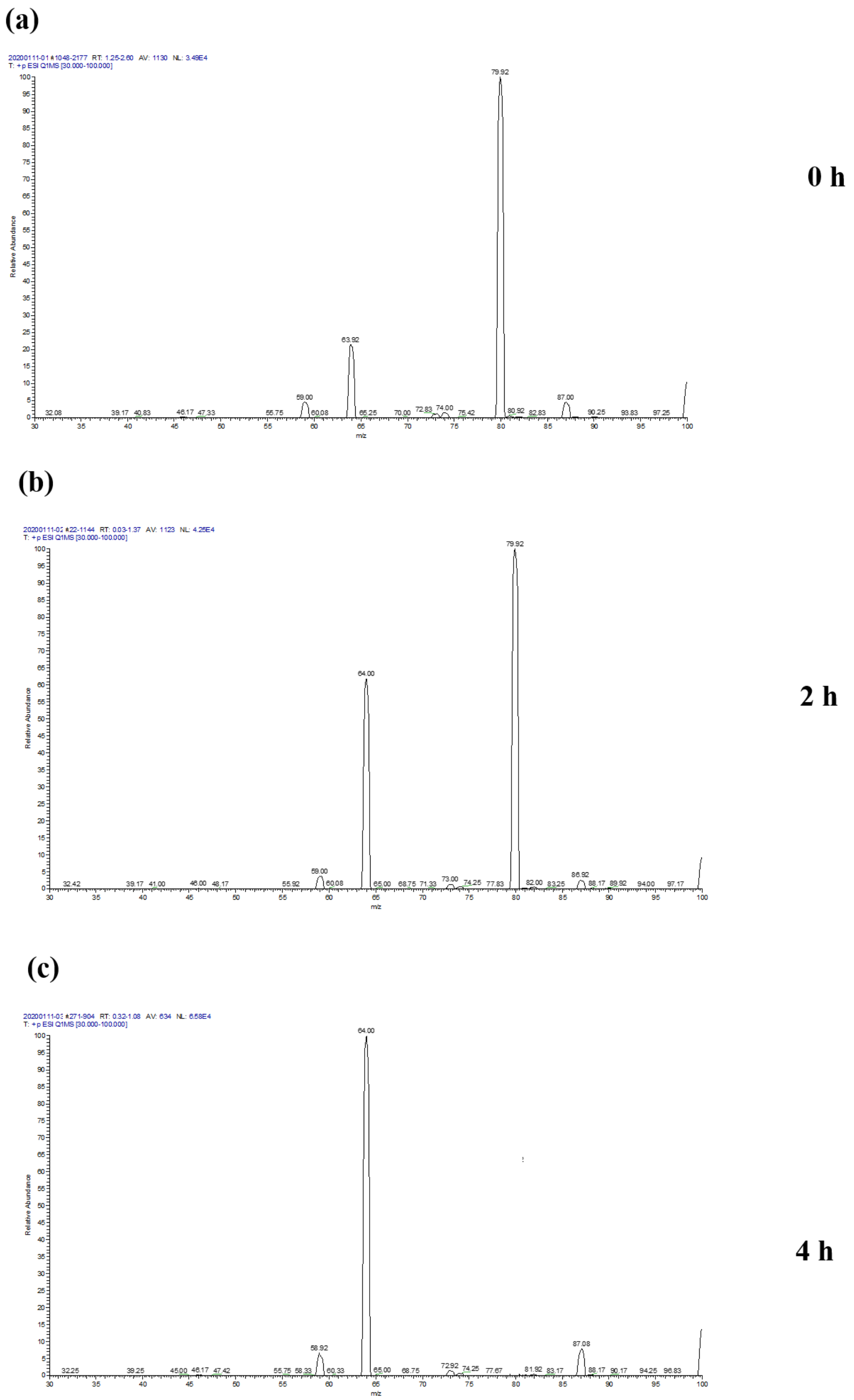

Figure S5. HPLC-MS spectrogram of pyridine degradation over 20%SnO_2@MCr composites at different reaction times: (a) 0 h, (b) 2.0 h, (c) 4.0 h.
